# Rare tumours of the pancreas: monocentric study

**DOI:** 10.1007/s00432-024-05884-2

**Published:** 2024-07-13

**Authors:** Astrid Bauschke, Annelore Altendorf-Hofmann, Aladdin Ali-Deeb, Michael Ardelt, Felix Dondorf, Falk Rauchfuss, Oliver Rohland, Aysun Tekbaș, Utz Settmacher

**Affiliations:** 1 Department of General, Visceral and Vascular Surgery, University Hospital Jena, Am Klinikum 1, 07740 Jena, Germany; 2 Comprehensive Cancer Center Central Germany (CCCG), 04103 Leipzig, Germany

**Keywords:** Rare epithelial pancreatic tumours, Long-term survival, Surgery

## Abstract

**Purpose:**

The biology of rare pancreatic tumours, which differs from that of ductal pancreatic cancer, requires increased attention. Although the majority of rare pancreatic tumours are benign, it is difficult to decide whether an invasive component exists without complete removal of the lesion, despite considerable progress in diagnosis. We are investigating a large cohort of patients with histologically confirmed epithelial non-ductal non-neuroendocrine neoplasms of the pancreas.

**Methods:**

Here we analyze long-term survival from patients, who underwent resection of histologically confirmed epithelial non-ductal non-neuroendocrine neoplasms of the pancreas. At our department between Jan 1st, 1999, and Dec 31st, 2019. The median follow-up was 61 (range 0–168) month. All statistical analyses were performed using SPSS 26.0 (IBM, Chicago, IL, USA) software.

**Results:**

46 patients (48%) were followed up for more than 5 years, 18 patients (19%) for more than 10 years. The 5-year and 10-year survival rates for rare non-invasive pancreatic tumours were 72% and 55% respectively. The proportion of rare tumour entities (non-ductal and non-neuroendocrine) increased continuously and statistically significantly (p = 0.004) from 4.2 to 12.3% in our clinic between 1999 and 2019. If there is no invasive growth yet, there is a varying risk of malignant degeneration in the course of the disease. Therefore, the indication for pancreatic resection is still the subject of discussion.

**Conclusion:**

The long-term prognosis of rare epithelial pancreatic tumours after R0 resection—even if they are already malignant—is much better than that of ductal pancreatic cancer.

## Introduction

A suspicious malignant mass in the pancreas is usually suspected to be a ductal adenocarcinoma. As a result of constantly improving cross-sectional imaging diagnostics and the almost universal availability of high-performance equipment, it is not only ductal pancreatic carcinomas that are being detected with increasing frequency. However, there is no correlation between the increasing detection of cystic pancreatic lesions and pancreatic malignancies (Singh et al. 2023). Furthermore, these lesions occur more frequently at an advanced age. Rare tumours are often seen unexpectedly during abdominal imaging by ultrasound, computer tomography or magnetic resonance imaging and are also increasingly being presented to the surgeon. These tumours of undetermined malignancy conceal a variety of morphological diagnoses, often with cystic components. Cystic neoplasms are divided into mucinous cystic neoplasms, solid pseudopapillary neoplasms, intraductal papillary mucinous neoplasms (IPMN) and serous cystic neoplasms. Furthermore, cystic neuroendocrine tumours can be observed. Cystic tumours can be differentiated on the basis of the age of the disease, gender, localisation in the pancreas, contact with the pancreatic duct and concentration of carcinoembryonic antigen (CEA) as well as lipase activity in the cystic fluid. The malignant potential of these masses varies (Buerlein and Shami 2021; European Study Group on Cystic Tumours of the 2018). “Worrisome features” or “high-risk stigmata” as an indication of malignancy are defined as solid components > 3 or > 5 mm, a thickened cyst wall, an abrupt change in duct calibre, cysts > 3 cm, an increase in cyst size > 3 mm/year, an enlargement of the pancreatic duct > 1 cm, local lymphadenopathy and an elevated serum CA-19-9 value as well as cytological evidence of “high-grade” dysplasia (European Study Group on Cystic Tumours of the 2018). The indication for surgical resection of rare pancreatic tumours prior to the manifestation of malignancy can be difficult in individual cases (Del Chiaro et al. 2017; Tjaden et al. 2021). An interdisciplinary case discussion should always be held in order to perform oncological pancreatic resection at an early tumour stage (Salvia et al. 2021). Zelga et al. (2022) recommended that the number of worrisome features should be taken into account when determining the indication for pancreatic resection.

The aim of the retrospective analysis was to investigate survival after pancreatic resection for rare pancreatic tumours over a long observation period in a monocentric patient population.

## Materials/methods

All patients with a histologically confirmed primary pancreatic tumour between Jan 1st, 1999, and Dec 31st, 2019 were re-evaluated in our clinic's prospective tumour registry. The characteristics evaluated were age, gender, date of treatment, tumour morphology, malignancy, distant metastases, type of treatment, radicality of treatment and progression. Tumours without detailed histological classification (malignant neoplasms, carcinoma without further details) and tumours of the papilla Vateri were excluded. Rare epithelial tumours have been reclassified according to the 5th edition of the WHO classification for tumours of the digestive tract (Nagtegaal et al. 2020). Follow-up records included the most recent patients’ visit with a gastroenterologist, follow-up information from the tumour centre, the general practitioners, telephone interviews and the date of death.

Peri-operative and operative treatment were determined by the interdisciplinary pancreato-hepatobiliary tumour conference. The study in human subjects was conducted with approval of the local ethics committee (Reg.no.: 2021-2426 data) in accordance with national law and the Declaration of Helsinki of 1975 (in the current revised form).

### Surgical approach

Pyloric-preserving pancreatic head resection was performed according to the recommendations of the International Study Group of Pancreatic Surgery with standard lymphadenectomy. For malignancies of the corpus and cauda, extended pancreatic left resection, lymphadenectomy and splenectomy were performed. Standard lymphadenectomy was performed according to the recommendations of the International Study Group of Pancreatic Surgery (ISGPS), including lymph node locations (Tol et al. 2014).

### Statistical methods

All statistical analyses were performed using SPSS 26.0 (IBM, Chicago, IL, USA) software. Categorical variables were evaluated for independence using the Chi square test or Fisher exact test as indicated. Survival was calculated from the date of first diagnosis. Overall survival (patients’ death irrespective of the cause of death) was used as the endpoint for estimating prognosis. The median follow-up time was calculated using the reverse Kaplan–Meier method. Survival curves were created using the Kaplan–Meier method, and the log-rank test was used to assess differences in survival. Statistical significance was defined as a *p* value < 0.05 for all analyses.

## Results

Between Jan 1st, 1999, and Dec 31st, 2019 1145 patients with a primary pancreatic tumour underwent surgery, which was evaluated ex-post on the basis of histology. In total, there were 896 ductal adenocarcinomas, 153 neuroendocrine tumours and 96 tumours with a different morphology (rare). The proportion of rare tumour entities (non-ductal and non-neuroendocrine) increased continuously and statistically significantly (*p* = 0.004) from 4.2 to 12.3% between 1999 and 2019. This series of rare pancreatic tumours comprises 90 epithelial tumours and 6 others (2 sarcomas, 2 soft tissue tumours, and two malignant lymphomas each).

18 of the 90 rare epithelial tumours (20%) were malignant invasive, 63 benign, 9 showed high-grade dysplasia. Of the 18 malignant tumours, 2 (11%) had already led to distant metastases.

**Fig. 1:**
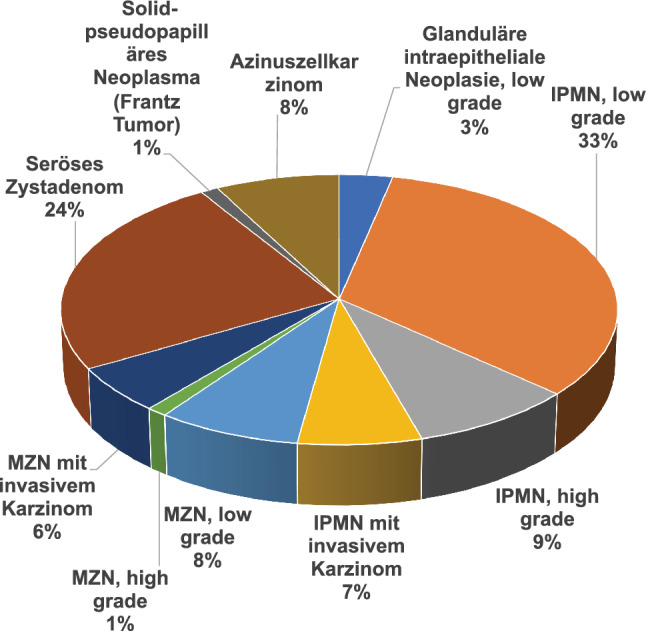
WHO Classification of Non-ductal non-neuroendocrine epithelial neoplasms of the pancreas

57 tumours (63%) were mucinous non-ductal neoplasms, 44 of which were intraductal papillary mucinous neoplasms (IPMN) and 13 mucinous cystic neoplasms (MCN) (Fig. 1).

All non-invasive tumours were radically resected. Of the 18 invasive tumours, 3 (17%) were no longer resectable. A biliodigestive anastomosis was created only once. The 15 other invasive tumours were radically resected.

A partial pancreatectomy was performed 65 times and a total pancreatectomy 19 times. 55 patients underwent open surgery, 10 minimally invasive. A surgical robot (Da Vinci) was used for 6 left-sided pancreatic resections. 77 of the 84 pancreatic resections (92%) were completed as R0 resections.

The patients affected by the various types of tumour differ in terms of age, gender distribution and localisation of the lesion in the pancreas. The most important characteristics of the various neoplastic tumours are summarised in Table 1.

**Table 1:** Patients under study

WHO Classification of rare pancreatic Tumour	Number	Age	Proportion of women %	Location head %
Glandular intraepithelial neoplasms, low grade	3			
Intraductal papillary mucinous neoplasm, low grade	30	67	50	50
Intraductal papillary mucinous neoplasm, high grade	8	73	33	50
Intraductal papillary mucinous neoplasm, with invasive carcinoma	6	76	67	50
Mucinous cystic neoplasm, low grade	7	61	100	29
Mucinous cystic neoplasm, high grade	1	60	0	100
Mucinous cystic neoplasm with invasive carcinoma	5	60	60	40
Serous cystadenoma	22	64	68	36
Solid pseudopapillary neoplasm (Frantz tumour)	1	24	100	Pancreatic tail
Acinar cell carcinoma	7	73	43	86
	90			

Low-grade glandular intraepithelial neoplasm (PanIN-1) is a change in the pancreatic duct without its own disease value. If it appears as a mass in the pancreas, it may nevertheless give rise to further diagnostics and—if malignancy is still suspected—to a pancreatic resection.

**Fig. 2:**
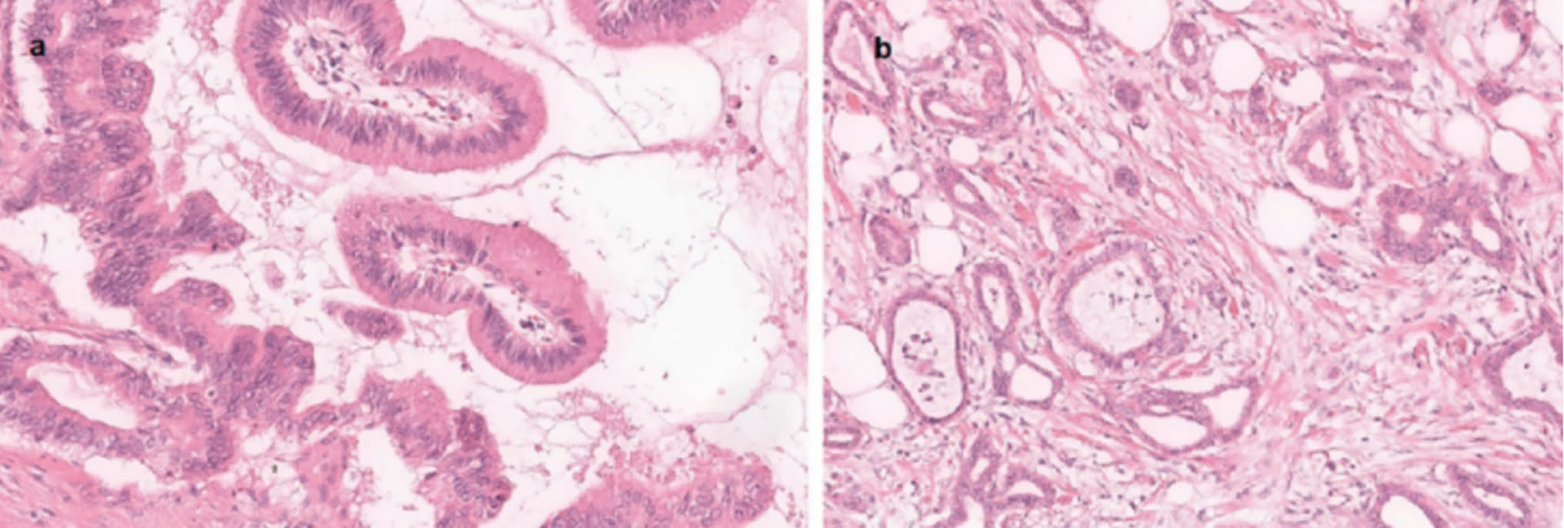
Histological representation of intraductal papillary mucinous neoplasms **a**: low grade (right) and high grade (left), **b**: invasive carcinoma

The IPMN were the most common tumour entity in our study. The malignant potential of IPNMs depends on their localisation in the ductal system. Of 30 tumours in the main duct, 11 (37%) were high grade or invasive, of the 12 in secondary ducts only one (9%) was (Fig. 2). Half of the 44 IPMNs were located in the pancreas head. 42 of the 44 IPMNs (96%) were resected. In the case of IPMN, an increase in patient age of 10 years is noticeable between the low-grade tumours and those with invasive carcinoma. Of a total of 44 IPMN, 42 were completely resected. 6 of the 44 IPMNs (14%) already showed invasive components.

**Fig. 3:**
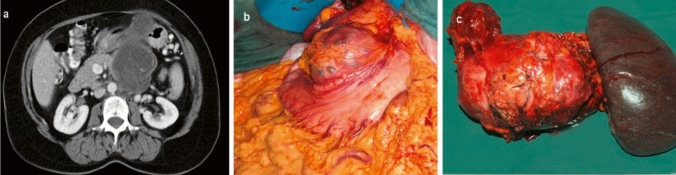
Mucinous cystic neoplasiapT3, pN0 (0/52), L0, V0, R0, G2. **a** Preoperative computed tomography, **b** intraoperative findings, **c** surgical specimen after subtotal pancreatectomy and splenectomy

15% of rare pancreatic neoplasms were mucinous cystic neoplasms (MCN). In terms of developmental history, these originate from ovarian systems. The unilocular lesion shows a closed capsule with an epithelial lining. 10 of the 13 mucinous cystic neoplasms (77%) were observed in women in our study. The lack of connection to the ductal system is typical (Fig. 3).

We only saw a serous cystic neoplasm as a serous cystadenoma. We were not able to observe a serous cystadenocarcinoma. Two-thirds of the cystadenomas occurred in women and predominantly in the pancreas body or tail.

We saw a young patient with a solid pseudopapillary neoplasm (SPN, Frantz tumour).

In our study, patients with acinar cell carcinoma were medianally younger than patients with adenocarcinoma and could also be resected more frequently (resection rate 39 vs. 11%).

### Follow-up

**Fig. 4:**
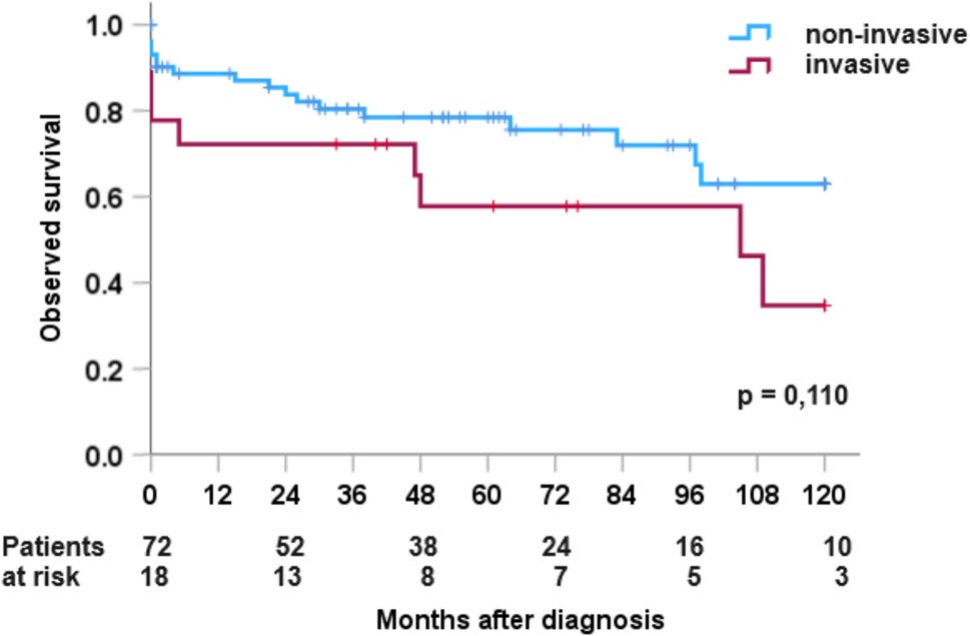
Survival of rare pancreatic tumours invasive versus non-invasive

Four of our patients developed liver metastases despite the initial diagnosis of a “non-invasive” pancreatic tumor and died from this tumor recurrence. 46 patients (48%) were followed up for more than 5 years, 18 patients (19%) for more than 10 years. After a median follow-up of 61 (range 0–168) months, 26 of the 87 patients with rare epithelial tumours had died. The three unresected patients died of their malignant tumour within 6 months of diagnosis. Of the 15 resected patients with malignant rare epithelial tumours, 5 died of carcinoma. Two patients died perioperatively, two from tumour recurrence and one patient died tumour-free at the age of 82 years, 105 months after R0 pancreatic head resection. The 5-year and 10-year survival rates for rare non-invasive pancreatic tumours were 72% and 55% respectively. For invasive pancreatic tumours, the rates were 54% and 39% (Fig. 4).

## Discussion

A large number of these space occupying lesions are being diagnosed in symptom-free patients with tomographic imaging for various indications and necessitate further diagnostics.

Multi slice CTs are widely available; they reduce artefacts and allow for multi plane reformation in selected imaging directions (axially, sagittally, coronally) with low slice thickness without loss of resolution with detailed 3D reconstruction. Thus, early diagnosis with differentiation of the cysts (solid, cystic), location within the pancreas, connection with the pancreatic duct and differential-diagnosis clarification of small space occupying lesions, is possible, too.

Symptoms of the patient, if present, do not help in the diagnosis of rare epithelial pancreatic tumours. In the Italian review, 85% of patients with cystic pancreatic tumours were asymptomatic (Pezzilli et al. 2020). Likewise, tumour marker levels (carbohydrate antigen 19–9, CEA) in the peripheral blood are also of little help. MRI with magnetic resonance cholangiopancreatography (MRCP) is superior to computed tomography (CT) in terms of identification of the pancreatic duct, mural nodules and multifocal lesions. A contrast enhanced (EUS) combined with a fine needle biopsy can detect malignant cells, determine the CEA and amylase value and thus contribute to therapy planning (Brugge et al. 2004). Diagnostic procedures have evolved significantly over the past years. Via EUS based fine needle biopsy the assessment of bio markers (CEA, amylase, lipase, VEGF-A, trypsine, chymotrypsine, catenin, E-cadherin, CK, AAT etc.) is possible in addition to histological markers and viscosity. This allows for better identification of patients with risk for aberration as opposed to those who do not require surgery with surveillance only (Mormul et al. 2023)*.* However, a reliable diagnosis can only be made by a pathologist after processing the pancreatic resectate.

There are guidelines from various professional societies on the therapy regimen for cystic pancreatic neoplasms: the guidelines of the International Association of Pancreatology (IAP), published in 2006 and updated in 2012 and 2016; the European evidence-based guidelines published in 2013 and updated in 2017; and the guidelines of the American Gastroenterological Association (AGA), published in 2015 (Elta et al. 2018; European Study Group on Cystic Tumours of the 2018; Tanaka et al. 2017).

When determining the indication for resection, it should be noted that the proportion of pancreatic resection without evidence of malignancy in the literature was 25–64% for cystic pancreatic lesions and up to 78% for mucinous pancreatic lesions (Litchinko et al. 2020; Perri et al. 2020; Springer et al. 2019). The lack of histological confirmation and thus the risk of misdiagnosis and malignant degeneration are the disadvantages of large observational studies (Han et al. 2018; Pergolini et al. 2017).

If surgical removal of cystic tumours is indicated, the risk of perioperative complications, mortality and impairment of quality of life, the substitution of pancreatic enzymes and any insulin medication after pancreatic resection must be weighed against the risk of malignant degeneration that is already present or occurs during the course of the procedure. When deciding against tumour resection, in addition to the delayed diagnosis of malignancy, the emotional strain on the patient as a result of regular follow-up and the costs of regular sectional imaging checks must be taken into account (Fong et al. 2023).

But even after radical tumour removal, there is a risk of recurrence or metastasis. Despite the most careful preparation of the specimen, the pathologist may overlook small nests of high-grade dysplasia or islet invasive growth, which then lead to local recurrence or metastases. In our study, three patients developed a recurrence after resection of a benign pancreatic tumour.

The minimally invasive procedure for precancerous pancreatic lesions improves compliance.

In several reviews and meta-analyses, minimally invasive and robot-based operations were technically equivalent to open surgery (Aiolfi et al. 2021; Zhang et al. 2021). However, parenchyma-sparing procedures such as enucleations and middle pancreatectomy should only be reserved for selected cases (European Study Group on Cystic Tumours of the 2018; Tanaka et al. 2017).

Mucinous non-ductal neoplasms of the pancreas were first described in 1978 and in 1993 differentiated into the entities of intraductal papillary mucinous neoplasms (IPMN) and mucinous cystic neoplasms (MCN) (Compagno and Oertel 1978; Doulamis et al. 2016; Hui et al. 2018). 14% (6/44) of our IPMNs already showed invasive growth. Sohn et al. (2001) found invasive growth in 37% (22/60). In Asian studies, the proportion of benign IPNM was usually around 50% (Hirono et al. 2020; Hong et al. 2022; Min et al. 2020). The malignant potential for main duct IPMN is reported to be 60–80% (Vege et al. 2015), in secondary duct IPMN, 3–26% (Vege et al. 2015). Of 30 tumours in the main duct, 11 (37%) were high grade or invasive, of the 12 in secondary ducts only one (9%) was. In our study, high-grade neoplasms were observed in the older patients. This underlines the importance of monitoring in older patients with risk factors and the performance of resection before the age of 65. In 2017, Del Chiaro et al. (2017) considered the development of a malignancy on the basis of an IPMN to be only a matter of time.

The risk of malignant disease in MCN is indicated by parameters similar to IPMN (cysts ≥ 4 cm, pancreatitis, diabetes, thickened cyst wall, solid cyst parts < 5 mm) (European Study Group on Cystic Tumours of the 2018; Kang et al. 2018; Keane et al. 2018; Ohtsuka et al. 2020; Postlewait et al. 2017). In the current literature, malignancy rates vary between 4 and 12% (Zhao et al. 2020). In the absence of malignancy criteria, a clinical follow-up is recommended. However, if risk criteria are detected, resection before formation of the malignant tumour is indicated (Marchegiani et al. 2021).

In contrast to mucinous tumours, serous cystic neoplasms very rarely degenerate (Nagtegaal et al. 2020). We have not seen any serous cystic adenocarcinoma. In the largest analysis by Jais et al. (2016) of 2622 patients, the initially conservative approach to serous cystic neoplasms is favoured. Nevertheless, there are case reports of serous cystic pancreatic neoplasms with aggressive local growth (Papazarkadas et al. 2022). Optimal management is the subject of controversial discussion. In general, observation is recommended for benign lesions. Surgical therapy should be reserved for symptomatic lesions or if malignancy cannot be excluded (Del Chiaro et al. 2013).

Solid pseudopapillary neoplasms (SPN, Frantz tumour) were first described by Frantz in 1959 (Frantz). So far, we have only seen one young woman with this tumour. She has survived pancreatic resection for more than 10 years without recurrence.

SPN are observed in 0.2 to 3% of pancreatic tumours, are often symptomless and occur predominantly in young women. They do not show any predominant localisation. The pathogenesis is undetermined. All SPN have a mutation in the β-catenin gene in common. The differential diagnosis of neuroendocrine tumours can be difficult in individual cases (Tasar and Kilicturgay 2022). There are a large number of studies on this rare tumour entity with long time intervals and a 5-year survival rate of > 90% (Table 2). Lymph node metastases are rarely observed (literature table). R0 resection, also in the case of simultaneous liver metastases or vascular resections, is of decisive importance for long-term survival (Liu et al. 2019). Given the young age of the patients, resection is recommended for the good prognosis of low-grade malignant tumours with a very good long-term prognosis (European Study Group on Cystic Tumours of the 2018).

**Table 2:** Literature review Solid pseudopapillary neoplasms of the pancreas

Solid pseudopapillary pancreatic tumour	Geography	Resected patient	Time period	N + %	R0 %	5-year overall survival all patients %
Marchegiani et al. (2016)*	Italy/USA	131	1986–2014	0.8	88	98 (DFS)
Beltrame et al. (2016)	Italy	18	1997–2013	5	100	100
Xu et al. (2017)	China	121	2007–2021	0.8	99	98
Lubezky et al. (2017)	Israel	32	1995–2016	0	100	96
Jutric et al. (2017)*	USA	296	1998–2011	12	86	85^#^
Huffman et al. (2018)	USA	282	2004–2012	n. r	81	98
Hanada et al. (2018)*	Japan	278	1990–2015	0.4	n. r	99
Tjaden et al. (2019)	Germany	52	2001–2018	3.8	89	89 (DFS)
Liu et al. (2019)	China	243	2008–2018	0.8	n. r	98
Li et al. (2022)*	China	133	2013–2019	6	93	99

*Multicentric, n. r. not reported, DFS desease free survival, ^#^8-year overall survival

It is worth noting that 10–15% of SPN show aggressive behaviour.

Acinar cell carcinomas of the pancreas are diagnosed in 1% of patients with pancreatic tumours (Chaudhary 2015). We only observed seven patients over the age of 70 with predominant tumour localisation in the head. The symptoms are usually atypical. There is no established marker similar to CA19-9 in adenocarcinoma (Zhou et al. 2020). Ki67, lymph node metastases and vascular invasion are relevant for prognosis (Xu et al. 2022). Early recurrences and metastases are observed in the small case series (Egal et al. 2019; Zong et al. 2020). Adjuvant therapy is based on adenocarcinoma.

The literature confirms the survival advantage after surgical therapy. In the analysis of the German Cancer Registry, patients with acinar carcinomas had a median age of 66 years. The resection rate was 56% and the R0 rate 53% (Petrova et al. 2021). As in other studies, we observed lymph node metastases in approx. 1/3 of patients and a 5-year survival rate of between 26 and 73% (Table 3).

**Table 3:** Literature review Acinar cell carcinomas of the pancreas

Author	Year	Geography	Resected patients	Time period	N + (%)	R0 (%)	5-year overall survival resected patients (%)
Schmidt et al. (2008)^b^	2008	USA (NCDB)	333 (39%)	1985–2005	32	64	36
Wisnoski et al. (2008)^a^	2008	USA (SEER)	266 (39%)	1988–2003	49	kA n.r	73
Landa et al. (2019)^b^	2019	USA (NCDB)	414 (42%)	1998–2012	35	81	42
He et al. (2018)^a^	2018	USA (SEER)	91 (< 40%)	2004–2014	n. r	n. r	≈40
Egal et al. (2019)*	2019	France	29 (66%)	2000–2018	27	79	≈60
Zong et al. (2020)^a^	2020	USA (SEER)	111 (36%)	2005–2018	n.r	n.r	37
Petrova et al. (2021)^c^	2021	Germany	131 (56%)	2000–2019	43	53	≈62
Sridharan et al. (2021)*	2021	USA	43 (65%)	1996–2019	n. r	n. r	≈35
Duorui et al. (2020)^a^	2021	USA (SEER)	75 (30%)	1996–2013	n. r	n. r	n. r
Bauschke	2023	Germany	6 (86%)	1999–2019	33	83	60

* Multicentric, ^a^SEER Surveillance, Epidemiology, and End Results, ^b^NCDB National Cancer Database, ^c^German Cancer Registry Group, n. r. not reported

## Conclusion

The biology of rare pancreatic tumours, which differs from that of ductal pancreatic cancer, requires increased attention. Although the majority of rare pancreatic tumours are benign, it is difficult to decide whether an invasive component exists without complete removal of the lesion, despite considerable progress in diagnosis. If there is no invasive growth yet, there is a varying risk of malignant degeneration in the course of the disease. Therefore, the indication for pancreatic resection is still the subject of discussion. Interdisciplinary cooperation on the tumor board is the decisive condition for treatment of rare pancreatic tumors, in addition to diagnostics including radiological tomographic imaging, endoscopy with fine needle biopsy and measurements of histological parameters for evaluation of the risk for malignant aberration as well as the necessary therapeutic procedures versus a watch-an-wait approach. There are discussions whether risky pancreatic resections with relevant early and late surgical morbidity, metabolic morbidity in symptom-free patients with a high proportion of benign entities are indicated. In our investigation, the therapeutic procedures were discussed on the interdisciplinary tumor board. The long-term prognosis of rare epithelial pancreatic tumours after R0 resection—even if they are already malignant—is much better than that of ductal pancreatic cancer.
